# Vitiligo: The Association With Metabolic Syndrome and the Role of Simvastatin as an Immunomodulator

**DOI:** 10.7759/cureus.14029

**Published:** 2021-03-22

**Authors:** Deepak Verma, Khadija Hussain, Karez S Namiq, Amena Firoz, Manel Bouchama, Maham Raza, Muhammad Haris, Safeera Khan

**Affiliations:** 1 Internal Medicine/Family Medicine, California Institute of Behavioral Neurosciences & Psychology, Fairfield, USA; 2 Radiology, California Institute of Behavioral Neurosciences & Psychology, Fairfield, USA; 3 Oncology, California Institute of Behavioral Neurosciences & Psychology, Fairfield, USA; 4 Pediatrics, California Institute of Behavioral Neurosciences & Psychology, Fairfield, USA; 5 Internal Medicine, California Institute of Behavioral Neurosciences & Psychology, Fairfield, USA; 6 Internal Medicine, Royal Lancaster Infirmary, Lancaster, GBR

**Keywords:** metabolic syndrome, vitiligo, dyslipidemia, hypertension, obesity, simvastatin

## Abstract

Vitiligo is an autoimmune condition primarily affecting the skin where there is destruction of melanocytes characterized by pinkish-white patches on the skin. It is associated with other autoimmune diseases such as thyroid disease, rheumatoid arthritis, diabetes mellitus, and metabolic syndrome. Metabolic syndrome is a constellation of disorders including insulin resistance, hypertension, dyslipidemia, and obesity, and is considered a leading cause of cardiovascular morbidity. Simvastatin is a potent hypolipidemic drug that also possesses immunomodulating properties and is a common drug used in dyslipidemia and cardiovascular diseases. This study aimed to assess the association between vitiligo and metabolic syndrome and explore the immunomodulating properties of simvastatin for use in vitiligo.

We reviewed various articles from PubMed, ResearchGate, and Google Scholar using different keywords and Medical Subject Headings and finalized 33 studies to be used in our review. The articles selected showed a positive association between vitiligo and metabolic syndrome or one of the component diseases of metabolic syndrome. The benefits of using simvastatin were also addressed by few articles attributing to its antioxidant and immunomodulating effect. However, there was no concrete explanation justifying the association between vitiligo and metabolic syndrome due to a limited number of studies and smaller sample size. Large-scale clinical trials should be conducted to evaluate the use of simvastatin as an immunomodulator in vitiligo to prevent possible metabolic complications.

## Introduction and background

Vitiligo is an acquired pigmentation disorder of the skin in which there is destruction of melanocytes. It is characterized by white patches on the skin. Around 0.1-2% of the world’s population is affected by the disease, irrespective of race and sex [1]. The disorder has been reported to have a high incidence in the second and third decade of life [1].

Vitiligo is classified into segmental and non-segmental vitiligo [2]. Non-segmental vitiligo is usually bilateral and symmetrical in distribution. Segmental vitiligo is unilateral and focal in distribution [2]. The etiopathogenesis of vitiligo involves various factors, which include oxidative stress [3], autoimmune destruction of melanocytes, neural hypothesis through an accumulation of a neurochemical substance that decreases melanin production, and sympathetic nervous system activity through direct cytotoxic effect and indirectly through the generation of free radicals [2-4].

The clinical course of vitiligo can be assessed using the Vitiligo Area Severity Index (VASI) and Vitiligo Disease Activity Score (VIDA) score. In general, vitiligo patients lead a normal healthy life. Still, some patients are at an increased risk of developing various systemic diseases such as thyroid disease, Addison’s disease, systemic lupus erythematosus, rheumatoid arthritis, diabetes, and metabolic syndrome [5].

A metabolic syndrome is a group of disorders related to the body’s metabolism, including diabetes, hypertension, obesity, and dyslipidemia. The National Cholesterol Education Program Adult Treatment Panel III defined metabolic syndrome as the presence of any three of the following five characteristics [6]:

1. Abnormal obesity defined as waist circumference of 102 cm in males and 88 cm in females.

2. Serum triglyceride level of 150 mg/dL or drug treatment for elevated triglyceride.

3. Serum high-density lipoprotein (HDL) cholesterol level of <40 mg/dL in males and <50 mg/dL in females or drug treatment for low HDL cholesterol.

4. Blood pressure of 130/85 mmHg or drug treatment for elevated blood pressure.

5. Fasting plasma glucose level of 100 mg/dL or drug treatment for elevated blood glucose.

**Figure 1:**
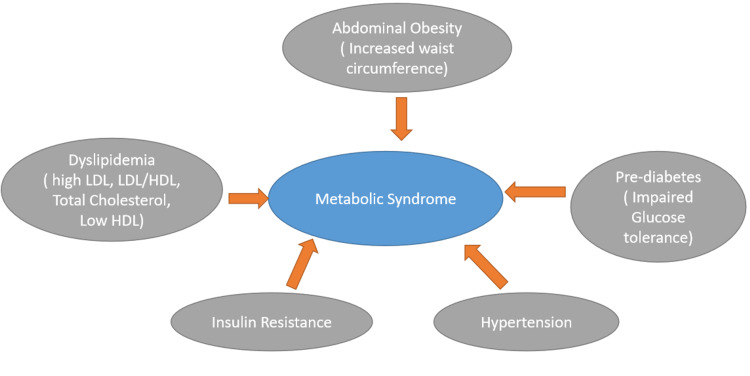
Risk factors associated with metabolic syndrome. LDL: low-density lipoprotein; HDL: high-density lipoprotein

While insulin resistance is believed to be the major factor involved in developing metabolic syndrome [7], oxidative stress has been suggested to cause the disorder by some studies [3]. Metabolic syndrome has become increasingly prevalent, with one-quarter of the adult population being affected [1]. Metabolic syndrome is associated with an increased risk of myocardial infarction, stroke, and diabetes [7]. Figure 1 depicts the risk factors associated with metabolic syndrome.

Simvastatin is a potent statin drug that inhibits 3-hydroxy-3-methylglutaryl coenzyme-A (HMG-CoA) reductase enzyme involved in cholesterol synthesis in the liver. It is a widely used drug for the treatment of dyslipidemia and cardiovascular diseases [8]. Isoprenoids are formed during cholesterol biosynthesis, which is known to induce inflammation via the intracellular second messenger system. Simvastatin, by inhibition of HMG-CoA reductase, helps prevent the generation of isoprenoids [9]. In addition, it directly inhibits CD8+ T-cells specific to melanocytes, which leads to limited proliferation and decreased interferon gamma (IFN-γ) production [10]. We will further try to analyze whether simvastatin can be used as an immunomodulating agent in vitiligo.

Some studies have shown the association between vitiligo and diabetes or hypertension or dyslipidemia based on smaller sample size. This systematic review explores the association between vitiligo and metabolic syndrome and explores the possibility of using simvastatin as an immunomodulator in vitiligo to prevent metabolic complications.

## Review

Method

Electronic Search

We followed the Preferred Reporting Items for Systematic Review and Meta-Analysis (PRISMA) guidelines for conducting our review [11]. We systematically searched PubMed, ResearchGate, and Google Scholar for relevant studies. Studies published from inception to 2021 were identified. The keywords metabolic syndrome, vitiligo, dyslipidemia, hypertension, obesity, and simvastatin were used either alone or in combination to search the articles. A manual search was also performed by going through the reference list of a few articles.

Inclusion and Exclusion Criteria

There was no language barrier. Articles published in English, Turkish, and Chinese were included. The studies that met the following criteria were included: (a) provided information regarding the association between vitiligo and metabolic syndrome, and (b) explained about the associated factors common to both disorders. Exclusion criteria were animal trials, duplicate data, irrelevant information to any of the disorders, or insufficient information.

Data Extraction

**Figure 2:**
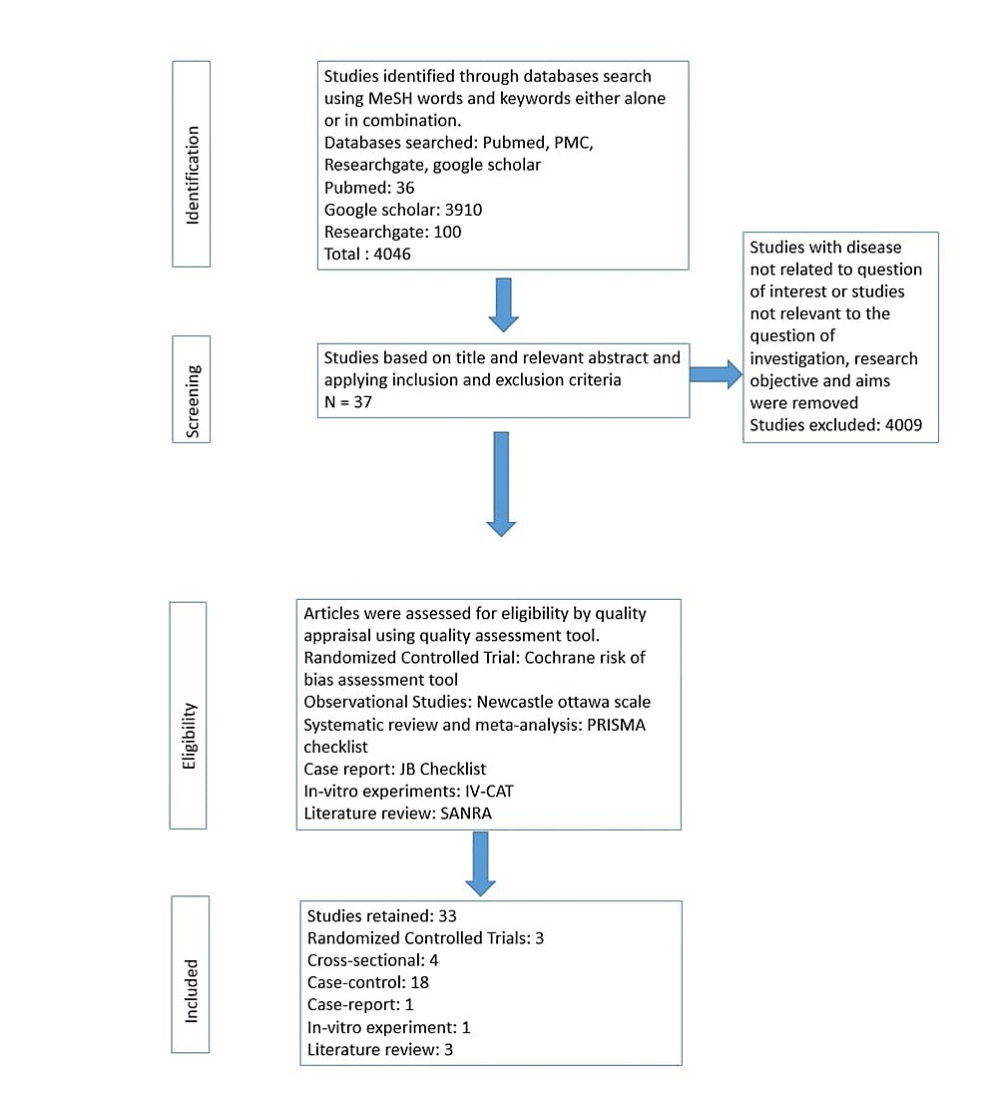
PRISMA flow diagram showing the methodology. PMC: PubMed central; PRISMA: Preferred Reporting Items for Systematic Reviews and Meta-Analysis; JB Checklist: Joanna Briggs Institute Checklist; IV-CAT: In Vitro Critical Appraisal Tool; SANRA: Scale for the Assessment of Narrative Review Article

Data were extracted using a standard data abstraction form. Relevant papers were reviewed independently, and any difference of opinions were resolved through mutual discussion. The following information was drawn out: first author’s name, year of publication, number of cases and controls, number of vitiligo patients having associated metabolic disorders, and number of patients participating in the clinical trials conducted. Figure 2 depicts the PRISMA flow diagram showing the methodology.

Results

A total of 4,046 articles were screened, of which 4,009 were excluded due to the following reasons: irrelevant information, not providing sufficient information, and duplicate data. A total of 37 articles fulfilled the inclusion criteria and were incorporated in our systematic review. We assessed 37 studies for quality using standardized quality assessment tools, and 33 articles were qualified for the quality appraisal. The following tools were used:

1. Randomized controlled trials: Cochrane Risk of the Bias Assessment Tool.

2. Observational studies: Newcastle-Ottawa Scale.

3. Case reports: Joanna Briggs Institute Checklist.

4. In-vitro experiments: In Vitro Critical Appraisal Tool.

5. Systematic reviews and meta-analyses: PRISMA checklist.

6. Literature review articles: Scale for the Assessment of Narrative Review Article.

Our review article included 1,315 vitiligo patients from 20 different observational studies, 1,600 patients of type 2 diabetes mellitus from two cross-sectional studies, 127,589 patients from three systematic reviews and meta-analyses, 44 patients from three clinical trials, and one patient from a case report.

Discussion

Various studies have shown the association between vitiligo and metabolic syndrome. While there have been various factors involved in developing both disorders, we will discuss a few areas that will help establish the association.

Role of Oxidative Stress

Oxidative stress has been a notorious factor involved in the development of various diseases, including vitiligo and metabolic syndrome, through the generation of reactive oxygen species (ROS). ROS plays a vital role in cellular events such as inflammatory response, cell growth alteration, and apoptosis [12]. Enzymatic and non-enzymatic antioxidants such as superoxide dismutase, glutathione peroxidase, and catalase are present in melanocytes whose primary function is to prevent oxidative damage. ROS such as superoxide ion and peroxide (H_2_O_2_) ion cause oxidative stress leading to the destruction of melanocytes via apoptosis [13]. In addition, melanocytes are found in adipose tissue, where they exert anti-inflammatory and antioxidant effects. Reduced number of melanocytes and melanogenesis leads to the development of metabolic syndrome through insulin resistance and lipid abnormalities [3].

Koca et al., in their study, showed excess free radical generation leading to peroxidative damage mediated by increased oxidative destruction of polyunsaturated fatty acid of phospholipids [14]. This oxidative destruction was supported by increased levels of malondialdehyde (MDA) and H_2_O_2_ [12,14,15]. Azzazi et al. and Khan et al. also found decreased total antioxidant capacity in patients compared to controls. They found a significant association between carotid intima-media thickness and MDA levels [12,15]. A study by Bhatti et al. mentioned that oxidative stress caused the deposition of ROS in cells and tissues, leading to mitochondrial dysfunction by interacting with mitochondrial and cellular components such as DNA, protein, and lipids [16]. Alteration in mitochondrial functioning has been implicated in the development of metabolic syndrome due to defective cell metabolism. Increased glucose level enhances ROS overproduction leading to morphological changes in mitochondria [16]. Accumulation of lipids and free fatty acids causes the insulin signaling pathway inhibition, which majorly contributes to metabolic syndrome [16]. Table 1 summarizes the role of oxidative stress.

**Table 1:** Role of oxidative stress. H_2_O_2_: hydrogen peroxide; MDA: malondialdehyde; CIMT: carotid intima-media thickness; TAC: total antioxidant capacity; HDL: high-density lipoprotein; TG: triglyceride; SOD: superoxide dismutase; GPx: glutathione peroxidase

Author	Year	Type of study	Number of patients	Purpose of study	Result	Conclusion
Ataş et al. [3]	2017	Case-control	63	To investigate the association of metabolic syndrome and vitiligo	Poor clinical features of vitiligo were found to be an independent factor for metabolic syndrome	Increased risk of developing metabolic syndrome in vitiligo
Azzazi et al. [12]	2020	Case-control	50	To determine the increased risk of cardiovascular events in vitiligo	Increased levels of H_2_O_2_ and MDA and decreased levels of TAC. Increased CIMT level	Increased risk of developing dyslipidemia and atherosclerosis, which increases the risk of developing cardiovascular diseases
Sinha et al. [13]	2019	Case-control	75	To assess the association of metabolic syndrome with vitiligo	A significant difference in HDL and TG in patients compared to controls	Metabolic syndrome was associated with vitiligo
Khan et al. [15]	2009	Case-control	30	To determine the level of antioxidants in patients	Increased levels of MDA, decreased levels of SOD, GPx, vitamin C, vitamin E, and TAC	Oxidative stress plays a vital role in melanocyte destruction leading to the development of vitiligo
Bhatti et. al. [16]	2017	Narrative review	-	To ascertain the role of mitochondrial dysfunction and oxidative stress in metabolic disorders	-	Mitochondrial dysfunctions are involved in aging, cancer, metabolic syndrome, and neurodegenerative disorders

Role of Pro-Inflammatory Cytokines and Neurochemical Hypothesis

It is a well-known fact that pro-inflammatory cytokines play a major role in developing various autoimmune diseases. Patients with active vitiligo have increased pro-inflammatory cytokines like tumor necrosis factor (TNF)-α, interleukin (IL)-6, IL-8, IL-1-β, IFN-γ, and some anti-inflammatory cytokines such as IL-5, and IL-10 [17]. Inflammatory cytokines are involved in the inhibition of the insulin signaling pathway by phosphorylation of serine residues of insulin receptor substrate-1, which leads to the development of insulin resistance in vitiligo. Karadag et al. showed that Insulin resistance in vitiligo developed due to cytokines or autoimmune reaction to melanocytes [18].

Pietrzak et al. showed that adipokines and TNF-α, IL-6, monocyte chemoattractant factor secreted from white adipose tissue during the process of glucose and lipid metabolism cause metabolic syndrome and cardiovascular diseases if any disturbance in their secretion occurs. They also showed that cytokines like TNF-α, IL-1, IL-6, and C-reactive protein (CRP) contributed in inducing insulin resistance and other metabolic complications. Process fostering lipid peroxidation that occurs in the epidermis and adipose tissue as well is a possible rationale of lipid abnormalities in vitiligo [19].

Another aspect that can be highlighted to demonstrate the association is the neurochemical pathway. It is a well-known fact that increased catecholamine and homocysteine levels are associated with an increased risk of hypertension, a constituent of metabolic syndrome. A study by Namazi et al. showed increased catecholamines in vitiligo patients compared to the control group, which they attributed to increased levels of stress in patients [2]. A study by Cucchi et al. found increased levels of catecholamines and their metabolites [20]. In contrast, Orecchia et al. also reported increased levels of catecholamines in patients with a shorter duration of disease, which was statistically not significant in their study [21]. Taneja et al., in their study, found elevated levels of LDL cholesterol, reduced levels of HD -cholesterol, increased LDL-to-HDL ratio, and increased levels of homocysteine. They concluded that the result of their study might be due to ongoing abnormal metabolic processes, and homocysteine may be a precipitating factor [22]. Increased homocysteine levels were also found in studies by Tsai et al. and Singh et al. correlating to disease activity [23,24]. Table 2 summarizes the role of cytokines and homocysteine.

**Table 2:** Studies reporting the role of cytokines and homocysteine. HTN: hypertension; ROS: reactive oxygen species; HOMA-IR: homeostatic model assessment of insulin resistance; LDL: low-density lipoprotein; HDL: high-density lipoprotein; SBP: systolic blood pressure; NE: nor-epinephrine; E: epinephrine; NMN: nor-metanephrine; MN: metanephrine; HVA: homovanillic acid; 5-HIAA: 5-hydroxyindoleacetic acid

Author	Year	Type of study	No. of patients	Purpose of study	Result	Conclusion
Namazi et al. [2]	2020	Case-control	83	To investigate the relationship between hypertension and vitiligo	Increased prevalence of HTN in vitiligo	Increased prevalence of HTN in vitiligo due to increased stress-causing release of catecholamines
Mitra et al. [17]	2017	Cross-sectional	51	To investigate the relationship between ROS and cytokines in patients with vitiligo	Generation of ROS was higher in patients with vitiligo along with increased levels of pro-inflammatory cytokines	Enhanced production of ROS-mediated lipid peroxidation, DNA damage, along with a decline in antioxidant capacity was accountable for disease progression
Karadag et al. [18]	2011	Case-control	57	To investigate the relationship between vitiligo and insulin resistance	Patients with vitiligo had elevated HOMA-IR, insulin, and C-peptide levels, increased LDL-to-HDL ratio, and reduced HDL cholesterol with an increase in mean SBP	Increased insulin resistance in vitiligo could be connected to mechanisms other than obesity like cytokine production or autoimmune reaction to melanocytes
Pietrzak et al. [19]	2014	Case-control	34	To assess lipid profile in vitiligo-affected children	Increased LDL, decreased HDL, and increased LDL/HDL and TG	Lipid disturbances in vitiligo patients were probably due to metabolic disturbances in adipose tissue and oxidative stress
Cucchi et al. [20]	2000	Case-control	70	To investigate the association between vitiligo and monoaminergic system	Levels of NE, E, NMN, MN, HVA, 5-HIAA were remarkably elevated in patients with vitiligo	Higher levels of catecholamines and metabolites indicate increased activity of the monoaminergic system, which might be due to stressful events
Orecchia et al. [21]	1994	Case-control	40	To investigate the relationship between vitiligo and monoaminergic system	HVA and NMN levels were increased. Both catecholamines and metabolites showed higher concentration in patients with a shorter duration of disease but were statistically not significant	Monoaminergic systems were unlikely to be related to vitiligo
Taneja et al. [22]	2020	Cross-sectional	54	To investigate homocysteine levels and lipid risk factors in vitiligo	Increased levels of LDL, homocysteine and LDL/HDL. Decreased level of HDL	Increased homocysteine levels may be a precipitating factor in the pathogenesis of vitiligo. Increased lipid levels may be due to abnormal metabolic processes
Tsai et al. [23]	2019	Review article	1,215	To investigate the role of homocysteine, folate, and vitamin B12 levels in vitiligo	Increased level of homocysteine, decreased level of B12, and folate levels not significantly raised	Vitiligo was associated with higher homocysteine and lower vitamin B12 levels, which correlate with disease activity
Singh et al. [24]	2011	Case control	30	To investigate the association between homocysteine levels and vitiligo	Increased homocysteine levels in patients with vitiligo	Increased homocysteine levels may be the cause for vitiligo in predisposed individuals

Clinical Outcomes

Metabolic syndrome results in a significant cardiovascular outcome, which is a widely studied aspect of the disorder. Ahmad et al. showed myocardial infarction as a possible outcome of vitiligo, which they attributed to elevated levels of IL-17 and decreased levels of vitamin D and calcium [7]. A study by Yuan et al. showed the prevalence of thyroid disorders in vitiligo such as overt hyperthyroidism and subclinical hyperthyroidism [25].

Biondi et al. showed that thyroid disorders were associated with various metabolic abnormalities including type 1 diabetes, type 2 diabetes, and metabolic syndrome. They explained that thyroid hormones affect food intake, resting energy expenditure, and consequently, lead to metabolic alteration [26]. Chang et al. showed a significant association of vitiligo with both type 1 and type 2 diabetes [27].

A study by Mubki et al. found a higher prevalence of increased fasting plasma glucose levels in patients with vitiligo, which was similar to the findings of another study by Gopal et al., who concluded that the prevalence of diabetes mellitus was statistically significant in patients with vitiligo [4,5]. A study by Al Houssein et al. found a significantly increased risk of diabetes and dyslipidemia and an increased risk of developing obesity and renal injuries in vitiligo patients [28]. Tanacan et al. also found an increased frequency of metabolic syndrome in patients with vitiligo [1]. Sharma et al., in their study, found the prevalence of impaired glucose tolerance and significantly high levels of triglyceride and low levels of HDL in patients than in controls [29]. Onan et al. found remarkably higher levels of insulin and triglyceride levels in patients with vitiligo. However, no significant increase was found in the prevalence of insulin resistance or metabolic syndrome in their study [30]. Increased prevalence of vitiligo in type 2 diabetes patients has also been seen in some studies [31,32].

Role of Simvastatin as an Immunomodulator in Vitiligo

Simvastatin, a potent statin drug used widely in dyslipidemia and cardiovascular diseases, also possesses antioxidant and anti-inflammatory properties by its action on CD8+ T cells. It inhibits the production of pro-inflammatory cytokines, which is an important factor in the pathogenesis of vitiligo, as discussed above [10]. A small clinical trial conducted by Vanderweil et al. found topical simvastatin to be more useful than the systemic form, citing potential adverse effects of the systemic form [33]. Zhang et al. found that oral simvastatin was safe to be used in vitiligo; however, their study concluded that the drug might not be effective in the treatment of vitiligo [34]. A case report by Noel et al. described repigmentation in a vitiligo patient by treating with a high dose of simvastatin, the effect of which they attributed to the immunomodulation by inhibition of CD8+ T cells in melanocytes by IFN-γ [35].

**Figure 3:**
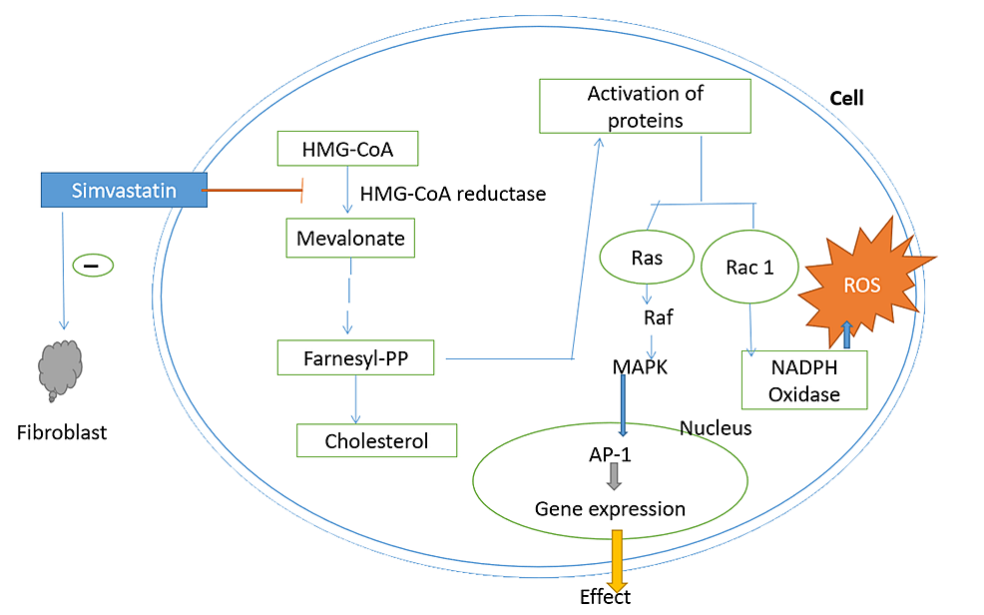
Mechanism of action of simvastatin. AP-1: activator protein; Farnesyl-PP: farnesyl pyrophosphate; HMG-CoA: 3-hydroxy-3-methylglutaryl coenzyme A; MAPK: mitogen-activated protein kinase; NADP: nicotinamide adenine dinucleotide phosphate; ROS: reactive oxygen species

Similarly, an in-vitro study by Chang et al. found that simvastatin was able to ameliorate H_2_O_2_-induced oxidative damage in human melanocytes by preventing the intracellular accumulation of ROS. They explained that it was probably due to the activity of antioxidant enzymes potentiated by the drug by activating nuclear factor erythroid 2- related factor, which contributed to mutual enhancement between mitogen-activated protein kinase pathway and p62. Their study showed that simvastatin protected cells from H_2_O_2_-induced cell apoptosis and ROS accumulation, highlighting the therapeutic potential of simvastatin in vitiligo [8]. It also blocks PI3k/Akt signal transduction pathway involved in T cell production. By virtue of these properties, patients with vitiligo can benefit from simvastatin as an immunomodulator and help prevent metabolic complications such as metabolic syndrome [9]. Figure 3 shows the mechanism of action of simvastatin.

Table 3 summarizes the role of simvastatin in treatment of vitiligo.

**Table 3:** Studies on the role of simvastatin in the treatment of vitiligo. RCT: randomized controlled trial; Nrf2: nuclear factor erythroid 2-related factor 2

Author	Year	Type of study	No. of patients	Purpose of study	Conclusion
Chang et al. [8]	2017	In-vitro experiment	-	To study the protective role of simvastatin against oxidative stress on human melanocytes	Defends against oxidative stress by stimulating Nrf2
Niezgoda et al. [10]	2019	RCT	24	To study the influence of simvastatin in vitiligo	-
Vanderweil et al. [33]	2017	RCT	15	To investigate the role of simvastatin in the treatment of vitiligo	Oral form may not be beneficial in the treatment of vitiligo
Zhang et al. [34]	2021	RCT	5	To assess the efficacy of simvastatin in the treatment of vitiligo	Oral simvastatin is safe but may not be effective in the treatment of vitiligo
Noel et al. [35]	2004	Case report	1	To assess the role of high-dose simvastatin as an immunomodulator in vitiligo	Simvastatin can be used as an immunomodulator in vitiligo

In our study, we came across the difference in studies collected for the review. Some studies directly established the association between vitiligo and metabolic syndrome, while other studies showed an association between vitiligo and one of the component disorders of metabolic syndrome. The other aspect of the study was that it could not ensure an established association between the disorders, which opens the possibility for more and better evaluation and research.

Limitations

Despite the availability of various resources and facilities for data collection, most of the articles were inaccessible either due to availability on a payment basis or institutional inaccessibility or citing confidentiality. Most of the data collected were observational studies based on a smaller sample size. Moreover, there was the unavailability of large randomized controlled trials or case-control studies regarding the use of simvastatin as an immunomodulator in vitiligo and its potential benefits in preventing metabolic complications. Our systematic review will hopefully be beneficial for further exploration on this topic.

## Conclusions

The data collected for the analysis of the association between vitiligo and metabolic syndrome showed hopeful results. Some studies addressed the association between the disorders, whereas others showed the association between vitiligo and one of the constituent disorders of metabolic syndrome, that is, insulin resistance or hypertension or dyslipidemia, instead of metabolic syndrome as a whole. Based on the reviewed articles, we found that some factors such as oxidative stress and pro-inflammatory cytokines play an important role in the development of both disorders. Oxidative stress leads to the generation of ROS, which destroys melanocytes via apoptosis. Reduced number of melanocytes in adipose tissue leads to insulin resistance and lipid abnormalities. Increased levels of cytokines lead to insulin resistance by phosphorylation of insulin receptors, which renders them resistant. Increased levels of homocysteine and catecholamines also contribute to the process. It is also noteworthy to acknowledge simvastatin’s role in preventing oxidative stress and cytokine-mediated complications in patients with vitiligo. However, this property of simvastatin is not much explored. Our systematic review will hopefully pave the way for the conduction of large-scale prospective studies to establish the relation between vitiligo and metabolic syndrome along with the direction of the association between the two and, hopefully, will help in exploring the benefits of simvastatin as an antioxidant therapy to prevent the development of metabolic complications.
